# When Prevention is Truly Better than Cure: Contrast-Associated Acute Kidney Injury in Percutaneous Coronary Intervention

**DOI:** 10.14797/mdcvj.1136

**Published:** 2022-09-06

**Authors:** Tea Isaac, Salima Gilani, Neal S. Kleiman

**Affiliations:** 1 Houston Methodist DeBakey Heart & Vascular Center, Houston Methodist Hospital, Houston, Texas, US

**Keywords:** coronary artery disease, contrast induced nephropathy, acute kidney injury, contrast associated kidney injury, percutaneous coronary intervention, cardiogenic shock

## Abstract

Contrast-associated acute kidney injury (CA-AKI) is a fairly frequent complication of cardiovascular angiography and percutaneous coronary intervention (PCI). The risk is significantly higher in patients with advanced chronic kidney disease (CKD). Prevention is the only option for avoiding the significant morbidity and mortality associated with CA-AKI. This review provides a concise and clinically directed appraisal of the latest pre-procedural and peri-procedural strategies to minimize the risk of CA-AKI in all patients undergoing PCI. By broadly implementing these evidence-based care bundles, we can dramatically improve outcomes in this vulnerable patient population.

## Introduction

The prevalence of chronic kidney disease (CKD) in patients with coronary artery disease (CAD) is high, with estimates of its prevalence ranging from 23% to 46%.^1,2^ Conversely, CKD is a major risk factor for CAD. As the glomerular filtration rate (GFR) declines to < 60 mL/min/1.73m^2^, the probability of developing CAD increases linearly.^3,4^ In fact, patients with stage 3 and 4 CKD have a mortality risk from cardiovascular disease that is approximately two to three times higher, respectively, than patients without CKD.^3,5^

As significant advances in percutaneous coronary intervention (PCI) have occurred, we now can perform increasingly complex cardiac interventional procedures that previously were not possible. Unfortunately, by the very nature of their complexity, such interventions frequently require large volumes of contrast and often occur in patients who already have established CKD.

In this manuscript, we aim to provide a concise and clinically useful appraisal of the latest pre-procedural and peri-procedural strategies to minimize the risk of CA-AKI in all patients undergoing PCI, with the hope that wide and more consistent implementation of these strategies can dramatically improve outcomes in this vulnerable patient population.

## Terminology

The association between contrast media and kidney dysfunction has been recognized for more than 80 years.^6^ In recent decades, the term “contrast-induced nephropathy” has been used to denote the development of acute kidney injury (AKI) following contrast administration in the absence of a plausible alternative etiology.^7^ Several recent revelations have indicated that this association is less robust than was previously accepted. For example, a study published in 2014 of 12,508 patients undergoing contrast-enhanced versus non–contrast-enhanced computed tomographic scans observed that the rate of AKI was no different between patients who did or did not receive contrast.^8^ Additionally, the investigators of AKI-MATRIX, a sub-study of the MATRIX (Minimizing Adverse Haemorrhagic Events by Transradial Access Site and Systemic Implementation of AngioX) trial, which compared radial versus femoral access for angiography and PCI in patients with acute coronary syndromes, reported a lower frequency of AKI among patients randomized to undergo radial catheterization despite similar contrast exposure to both groups.^9^ More recently, the term “contrast-associated acute kidney injury” (CA-AKI) has been adopted by some radiology and nephrology communities to refer to AKI that occurs shortly after administration of contrast.

### Definition of Contrast-Induced Nephropathy

There are several definitions for CA-AKI (Table 1). The most widely adopted is the Kidney Disease Improving Global Outcomes definition: an increase in serum creatinine by ≥ 0.3 mg/dL within 48 hours after contrast exposure or an increase to ≥ 50% within 7 days.^10^ These criteria also have been adopted by the National Cardiovascular Data Registry (NCDR).

**Table 1:** Definitions of contrast-associated acute kidney injury. KDIGO: Kidney Disease Improving Global Outcomes; NCDR: National Cardiovascular Data Registry-Acute Kidney Injury; AKIN: Acute Kidney Injury Network; RIFLE: Risk, injury, failure, loss of kidney function, and end-stage kidney disease; ESUR: European Society of Urogenital Radiology.


**KDIGO** **(also used by NCDR)**	Increase in serum creatinine of ≥ 0.3 mg/dL within 48 hours or ≥ 50% within 7 days, **or** urine output of < 0.5 mL/kg/hour for > 6 hours

**AKIN**	Increase in serum creatinine of ≥ 0.3 mg/dL or ≥ 50% within 48 hours, **or** urine output of < 0.5 mL/kg/hour for > 6 hours

**RIFLE**	**Risk:** Increase in serum creatinine to 1.5 times baseline, **or** urine output of < 0.5 mL/kg/hour for 6 to 12 hours **Injury:** Increase in serum creatinine of up to 2 times baseline, **or** urine output of < 0.5 mL/kg/hour for 12 to 24 hours **Failure:** Increase in serum creatinine to 3 times baseline, **or** increase in serum creatinine by > 0.5 mg/dL to > 4.0 mg/dL, **or** urine output of < 0.3 mL/kg/hour for > 24 hours or anuria for > 12 hours, **or** initiation of kidney replacement therapy **Loss:** Need for kidney replacement therapy for > 4 weeks **End stage:** Need for kidney replacement therapy for > 3 months

**ESUR**	Increase in serum creatinine by more than 25% or 0.5 mg/dL within 3 days following the intravascular administration of a contrast medium in the absence of an alternative etiology


## Epidemiology and Prognosis of Contrast-Induced Nephropathy

CA-AKI is an underdiagnosed complication of coronary angiography and PCI that has been associated with increased in-hospital morbidity and mortality, increased length of stay, and health care expenditure.^11,12,13,14^ A study of 985,737 patients enrolled in the NCDR reported that CA-AKI occurred in 7.1% of patients undergoing PCI.^15^ Often, estimates have ranged from 2% in patients with normal baseline kidney function to as high as 20% to 30% in patients with underlying advanced CKD.^13,14^ It should also be noted that as same-day discharge after PCI becomes more common, the frequency with which acute kidney injury is reported is likely to decrease since the rise in serum creatinine often occurs several days after contrast exposure. Fortunately, the incidence of CA-AKI requiring dialysis following PCI is rare.^16^ Importantly, both transient and persistent post-procedural renal dysfunction are independent predictors of mortality.^17^ Multiple reports have indicated that 1-year and 5-year estimated mortality rates are significantly higher in patients with CA-AKI^14^ and even more if dialysis was required.^13^

Recently, Mohebi et al. sought to investigate the determinants and outcomes of patients with CA-AKI from the ADAPT-DES (Assessment of Dual Antiplatelet Therapy with Drug Eluting Stents) registry. They included 7,412 patients with creatinine measured before and within 3 days after PCI. CA-AKI was defined as an absolute increase in creatinine > 0.5 mg/dL or 25% increase over baseline. As in other studies, multivariate analysis confirmed that patients developing CA-AKI were older, more acutely ill, more likely to be female, and to have diabetes and underlying CKD. Procedural variables associated with CA-AKI included hypotension during PCI, PCI for ST elevation myocardial infarction (STEMI), radial access, greater number of stents (a surrogate for contrast volume), and use of an intra-aortic balloon pump. At 2 years, CA-AKI was associated with a 2-fold increase in cardiac death and an increased risk of myocardial infarction, stent thrombosis, and bleeding, with the highest risk seen in the subgroup of patients with underlying CKD (estimated GFR < 60 mL/min/1.73m^2^) who went on to develop CA-AKI.^18^ Importantly, even in patients with normal renal function, development of CA-AKI increased the risk of net adverse clinical event (NACE) and mortality.

## Pathophysiology of Contrast-Associated Acute Kidney Injury

The pathophysiology of CA-AKI is complex and poorly understood. Changes in the renal circulation through contrast-mediated release of endothelin and adenosine with a simultaneous reduction in the availability of nitric oxide and prostaglandins is thought to be a major contributor. This series of events ultimately causes vasoconstriction and decreases renal perfusion, leading to ischemic and hypoxic damage to the renal medulla (especially vulnerable to ischemia) and the tubular epithelium.^7,19,20,21^

In addition, iodinated contrast also has direct toxic effects on the tubular epithelium. The increases in osmotic load and viscosity associated with high osmolarity contrast impairs blood oxygen delivery in the tubular epithelium, increasing hypoxia in the renal medulla.^22^ Medullary hypoxia can also trigger the release of reactive oxygen species, which cause additional tissue damage through oxidative stress.^7,22^

Finally, there are several peri-procedural factors, including hypotension, bleeding, and the inadvertent embolization of atheromatous debris, that amplify the deleterious effects of contrast media and compound the development of AKI.

## Risk Factors for Contrast-Associated Aki

There are multiple independent predictors of CA-AKI and dialysis, with each risk factor having varying contributions.^15^ These risk factors are best classified into modifiable and nonmodifiable risk factors. Modifiable risk factors may be patient or procedure related.

### Nonmodifiable Risk Factors

#### Age

Advanced age is a risk factor for CA-AKI. Some studies have found age ≥ 75 years to be an independent risk predictor of CA-AKI.^23,24^ The cause is likely to be multifactorial and includes age-related reduction in tubular function and GFR, renal atherosclerosis, and reduced regenerative capacity of the renal parenchyma following acute injury.

#### Chronic Kidney Disease

Preexisting CKD is the most important risk factor for the development of CA-AKI following PCI. Elevated serum creatinine at baseline is an independent predictor of CA-AKI.^15^ Similarly, patients with advanced CKD (estimated GFR [eGFR] < 30 mL/min/1.73m^2^) have more than a 3-fold increase in the adjusted risk of CA-AKI when compared to patients with normal renal function.^15^

#### Diabetes Mellitus

Diabetes is another independent risk factor for the development of CA-AKI and the subsequent need for dialysis, independent of preexisting renal dysfunction.^24,25^ Diabetes and CKD also appear to act synergistically. In patients who have both diabetes and CKD, the incidence of CA-AKI increases an additional 2-fold (compared to those with diabetes but without CKD) and can be as high as 33%.^26,27^

#### Cardiac Risk Factors

Acute decompensated heart failure (especially with left ventricular dysfunction), acute coronary syndrome, and cardiogenic shock have all been associated with an increased risk of CA-AKI.^14,24^ The overlying etiology here is related to renal hypoperfusion.

### Patient-Related Modifiable Risk Factors

#### Volume Depletion and Hemodynamic Instability

The risk of CA-AKI increases dramatically when the patient has reduced effective circulatory volume. This holds true regardless of the cause of hypovolemia, such as over diuresis, sepsis, or liver failure. Hypotension from cardiogenic shock and acute congestive heart failure also predispose patients to increased risk of CA-AKI.^15^

#### Anemia

Patients with CKD are more likely to have anemia (defined as hemoglobin < 12.0 g/dL in women and < 13.0 g/dL in men), which also worsens with declining renal function. Anemia itself has been shown to be an independent predictor of CA-AKI.^27^ Li et al. demonstrated that in patients undergoing PCI, the incidence of CA-AKI in those who are anemic is higher than that of non-anemic patents (6.3% versus 2.2%; *P* < .01). As GFR decreases < 60 mL/min, the incidence of CA-AKI in anemic patients may increase to 2-fold higher compared to that of non-anemic patients.^28^ The pathophysiology is likely related to exacerbation of local renal hypoxia following contrast media exposure in anemic patients.

#### Nephrotoxic Drug Use

Concomitant use of nephrotoxic agents such as nonsteroidal anti-inflammatory drugs, cyclosporine-A, tacrolimus, and diuretics increase the risk of CA-AKI by interfering with the autoregulatory responses of the renal circulation. Of note, the data regarding the need to discontinue angiotensin-converting enzyme inhibitors (ACEIs) prior to a procedure are conflicting, and data concerning angiotensin receptor blockers (ARBs) are even more limited.^29^ The latest data from the CAPTAIN (Angiotensin-Converting Enzyme Inhibitors and Contrast-Induced Nephropathy in Patients Receiving a Cardiac Catheterization) trial demonstrated that withholding ACEI/ARB ≥ 24 hours pre-procedure resulted in a nonsignificant reduction in CA-AKI and a significant reduction in post-procedure creatinine increase in patients who had moderate renal insufficiency (creatinine ≥ 1.7 mg/dL within 3 months) undergoing cardiac catheterization.^30^ Given the above, the most prudent approach would be to withhold ACEI/ARB preoperatively in patients with moderate CKD and/or deemed to be at high risk of CA-AKI, and to reinstitute therapy when appropriate. This is a low-cost intervention with significant potential benefits.

### Procedure-Related Modifiable Risk Factors

#### Timing of Angiography and Urgency of Procedure

The timing of cumulative renal injury related to contrast media exposure plays an important role in the development of CA-AKI, even if PCI is not performed. In fact, patients undergoing coronary artery bypass graft surgery within 1 day of coronary angiography have been found to have an approximate two-fold increase in the risk of major adverse renal and cardiac events compared to those who waited ≥ 5 days.^31^

The risk of CA-AKI is also significantly higher in patients undergoing emergency PCI, for example primary PCI in STEMI, than among patients with chronic coronary syndrome undergoing elective PCI.^15,32,33^ This is likely due to a combination of impaired systemic perfusion from left ventricular dysfunction, possible use of larger volumes of contrast, and the lack of opportunity for CA-AKI prophylaxis given the emergent nature of the procedure.

However, when it comes to staged versus ad-hoc PCI, the benefit of staging is not as clear. Interestingly, Shah et al. showed that across all patients along the entire spectrum of GFR, CA-AKI was not less frequent when PCI was staged compared to when PCI was performed ad-hoc. While the decline in renal function observed in both groups did not differ significantly, worse renal outcomes at 30 days were observed in the staged PCI group with a baseline GFR < 60 mL/min/1.73m^2^. In these patients, staged PCI was associated with a 2.6-fold greater decline in renal function 4 to 12 weeks after the procedure. This adverse effect of staging the procedures might occur because staged PCI exposes patients to greater cumulative contrast loads or because patients at higher risk for kidney injury may be more likely to have the procedures staged. Additionally, the temporal separation between procedures (within 30 days) may not be adequate, leading to recurrent hits to the nephrons and hence added superimposed acute kidney injury.^34^

#### Volume of Contrast Media

Volume of contrast used is the primary modifiable risk factor. Ample evidence from multiple studies indicates that higher volume contrast use significantly increases the risk of CA-AKI and that its detrimental effects are more pronounced with more progressive CKD.^7,21,35^ A contrast volume to creatinine clearance (CV/CrCl) ratio > 2 is an independent predictor of CA-AKI in patients with an eGFR < 30 mL/min/1.73m^2^.^36^ The risk of CA-AKI is minimal in patients receiving less than 100 mL of contrast during procedures or if the volume of contrast used is less than 5 mL/kg/serum Cr.^25^

#### Osmolality of Contrast Media

Although common parlance categorizes iodinated contrast agents as being high, iso, or low osmolar, they are technically categorized according to their osmolality rather than osmolarity. The risk of CA-AKI is increased with high-osmolal contrast use, particularly in patients with CKD.^37^ As a result, we have now gravitated to using iso-osmolal or low-osmolal contrast media as a standard because iso-osmolal contrast is less arrhythmogenic and is associated with a lower risk of CA-AKI, particularly in patients with existing CKD.

#### Hemodynamic Support

There continues to be uncertainty about the value of mechanical circulatory support (MCS) in reducing the risk of CA-AKI in patents who are hemodynamically unstable. However, encouraging data indicate that the use of left ventricular assist devices, mainly Impella (Abiomed), to maintain hemodynamic stability and end-organ perfusion in high-risk PCI patients has a role in reducing the incidence of CA-AKI.

## Risk Assessment for Contrast-Associated Acute Kidney Injury

In patients with CKD, every effort should be made to perform an appropriate risk assessment prior to PCI so both the physician and patient understand the benefits and risks of the procedure. Multiple validated scores can be used to facilitate this risk assessment. These scores all work on the same principle using a combination of the risk factors that predispose patients to CA-AKI after PCI. The three major scoring systems include that proposed by Mehran et al.,^32^ the Blue Cross Blue Shield of Michigan Cardiovascular Collaborative model,^38^ and the National Cardiovascular Data Registry Cath-PCI registry AKI prediction model.^15^

The Mehran scoring system, originally published in 2000, is the most commonly used system for CA-AKI risk stratification and has been validated in a cohort of 8,357 patients undergoing PCI. Eight variables—including hypotension, intra-aortic balloon pump, congestive heart failure NYHA Class III/IV, age > 75 years, anemia, diabetes, contrast media volume, and eGFR—are weighted individually and then combined to create a score that stratifies patients into low (≤ 5) and high risk (≥ 16) for CA-AKI.^16^ An increasing risk score is strongly associated with CA-AKI, ranging from 8.4% to 55.9% for a low- to high-risk score, respectively. This score has been validated in groups undergoing both elective and emergent procedures for acute coronary syndrome (STEMI and non-STEMI, or NSTEMI).^39^

Mehran et al. recently demonstrated a more contemporary scoring system (the Mehran-2 score) that includes two models. Model 1 consists of the following clinical variables: clinical presentation, eGFR, left ventricular ejection fraction, diabetes, hemoglobin, basal glucose, congestive heart failure, and age. Model 2 goes a step further to include procedural variables: contrast volume, peri-procedural bleeding, no flow or slow flow post procedure, and complex PCI anatomy. The Mehran-2 score was able to discriminate the risk of CA-AKI accurately, although model 2 only slightly improved the discrimination of the risk score (C-statistic in the derivation cohort: 0.72 for model 1 and 0.74 for model 2; in the validation cohort: 0.84 for model 1 and 0.86 for model 2). More importantly, this study also demonstrated that patients with CA-AKI had significantly increased risk of death at 1 year (10.2% versus 2.5%; adjusted hazard ratio 1.76; 95% CI, 1.31-2.36; *P* = .002).^40^

## Pre-Procedural Strategies to Prevent Contrast Associated Acute Kidney Injury

When patients are deemed at high risk of CA-AKI and PCI is still the most appropriate intervention, aggressive CA-AKI prevention strategies should be instituted to reduce this risk. The single most important pre-procedural strategy to reduce CA-AKI after PCI involves intravascular volume expansion through hydration initiated prior to and continued after the procedure.^7,41,42^ Hydration should be performed with intravenous normal saline. Current data indicate there is no added advantage of other solutions, including sodium bicarbonate, mannitol, or half normal saline. Normal saline also remains the most cost-effective option.

In patients without significant comorbidities, intravascular volume expansion with intravenous normal saline is safe and effective. This issue becomes more complex in patients with congestive heart failure. Fortunately, the Prevention of Contrast Renal Injury with Different Hydration Strategies (POSEIDON) trial demonstrated the feasibility of tailoring hydration to reduce the risk of CA-AKI without compromising a patient’s euvolemic status. This study included 396 patients with CKD randomized to either hydration based on left ventricular end-diastolic pressure (LVEDP) (5 mL/kg/hr for LVEDP < 13 mm Hg, 3 mL/kg/h for LVEDP = 13-18 mm Hg, and 1.5 mL/kg/h for LVEDP > 18 mm Hg) or standard hydration (fluid at 1.5 mL/kg/h). Intravenous saline was administered before contrast exposure and continued for 4 hours after the procedure. The total volume of normal saline administered was higher in the LVEDP-guided hydration arm (1,727 mL vs 812 mL; *P* < .001). CA-AKI was seen in 6.7% of the LVEDP-based hydration group compared with 16.3% of the standard hydration group (RR 0.41; 95% CI, 0.22-0.79; *P* = .005).^43^ The 6-month rate of MACE (major adverse cardiovascular events, a composite of all-cause mortality, myocardial infarction, or renal replacement therapy) was also lower in the LVEDP-guided group (3.1% vs 9.5%, *P* = .008). Similar effectiveness of pressure-guided hydration also can be accomplished using right atrial pressures.^44^

There is no role for the intravenous administration of sodium bicarbonate. While it may seem intuitive that alkalization of urine could reduce contrast-induced generation of oxygen free radicals that cause oxidative damage to renal tubular cells, several trials and subsequent meta-analyses have not consistently shown a benefit of isotonic sodium bicarbonate when compared with normal saline in reducing CA-AKI.

Similarly, N-acetylcysteine (NAC) was thought to be protective by decreasing endothelial dysfunction and scavenging oxygen free radicals, hence reducing direct oxidative damage to the renal parenchyma. Initial trials, including APART (Acetylcysteine to prevent angiography-related renal tissue injury) and RAPPID (Evaluation of the National Randomized Proton Pump Inhibitor De-prescribing Program), showed promising results with a reduction in CA-AKI in patients treated with NAC.^45,46,47^ However, further definitive studies have since had inconsistent results and recent meta-analyses of multiple trials demonstrate only a nonsignificant trend towards benefit in patients treated with NAC.

**Figure 1:**
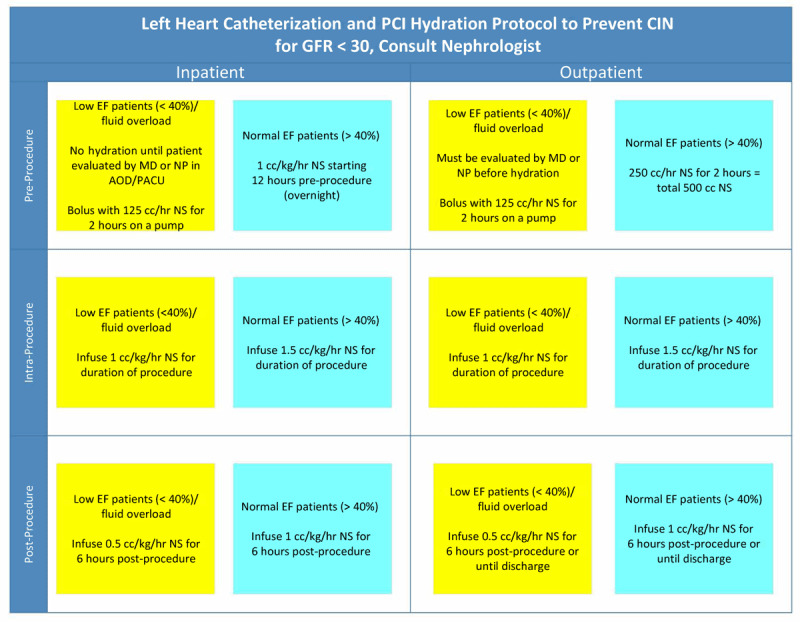
Houston Methodist Cardiac Catheterization Lab hydration protocol to prevent contrast associated acute kidney injury. EF: ejection fraction; PACU: post-anesthesia care unit; NS: normal saline

This has led to the Prevention of Serious Adverse Events Following Angiography (RESERVE) randomized trial, which provided conclusive evidence that there was no benefit of sodium bicarbonate and/or NAC over placebo among patients undergoing angiography who are at risk for CA-AKI.^48^ The incidence of CA-AKI was 9.5% in the sodium bicarbonate group and 8.3% in the sodium chloride group (OR 1.16; 95% CI, 0.96-1.41; *P* = .13). Similarly, the incidence of CA-AKI was 9.1% in the N-acetylcysteine group and 8.7% in the placebo group (OR 1.06; 95% CI, 0.87-1.28; *P* = .58).^48^ Accordingly, the recommended hydration protocol is the intravenous administration of normal saline at a rate of 1 to 1.5 mL/kg/hr for 3 to 12 hours pre-procedure, and 12 to 24 hours post-procedure—as per the Society for Cardiovascular Angiography and Interventions (SCAI) and European Society of Cardiology (ESC) guidelines^49,50^—with the option to tailor the aggressiveness of hydration to LVEDP or right atrial pressure where appropriate (Figure 1).

More recently, devices like the RenalGuard system (RenalGuard Solutions) show promise in further reducing the risk of CA-AKI after PCI. This device makes use of urine flow rate-guided hydration infusion to prevent CA-AKI. One hour before the procedure, an intravenous bolus of normal saline and a dose of furosemide are administered. PCI is started when a urine output of 300 mL/h is achieved. Similarly, matched hydration is continued for another 4 hours following PCI. The idea is that the high urine output dilutes the contrast media (in renal tubule urine) and allows its rapid elimination of contrast from the body, reducing its contact time within the nephrons and its deleterious effects on the kidney.

The REMEDIAL II (compared to standard hydration with sodium bicarbonate solution and NAC)^51^ and REMEDIAL III (compared to LVEDP-guided hydration)^52^ trials have shown the system to be superior in preventing CA-AKI compared to current therapies. In REMEDIAL III, CA-AKI for urine flow rate-guided versus LVEDP-guided angiography/PCI was 5.7% versus 10.3% (*P* = .036). Further clinical trial data are necessary to provide firm recommendations on this approach, but its use should be considered in patients deemed to be at high risk for CA-AKI if it is available.

Of note, Solomon et al. had demonstrated in 1994 that a forced diuresis regimen achieved by high-dose furosemide may be deleterious^47^ compared to hydration with half normal saline alone. However, significant weight loss was observed in the patients treated with furosemide, suggesting that the potentially deleterious effect of furosemide was the result of a negative fluid balance. The algorithm used with the RenalGuard system changes this paradigm by maintaining a constant intravascular volume to prevent hypovolemia while allowing a much higher total hydration volume when it is used (2,598 ± 1,349 mL in the UFR-guided group versus 1,709 ± 1,116 ml in the LVEDP-guided group; *P* < .001 in the REMEDIAL III study). This would account for the drastically different results demonstrated by Solomon et al.

HMG-Co A reductase inhibitors (statins) are believed to have beneficial pleiotropic effects that act to stabilize the renal vascular endothelium through their antioxidant and anti-inflammatory properties. The Patients with Renal Insufficiency Undergoing Coronary Angiography (PROMISS) trial showed no protective effect of simvastatin versus placebo in preventing CA-AKI. In contrast, the Rosuvastatin and Antiplatelet Therapy on Contrast-Induced Acute Kidney Injury and Myocardial Damage in Patients with Acute Coronary Syndrome (PRATO-ACS) trial demonstrated that high-dose rosuvastatin (40 mg loading dose followed by a 20 mg maintenance dose) when compared with placebo significantly reduced the risk of CA-AKI in statin-naive patients presenting with NSTEMI (6.7% versus 15.1%).^53^ Patti et al. also demonstrated similar benefits in the Atorvastatin for Reduction of MYocardial Damage during Angioplasty–Contrast-Induced Nephropathy (ARMYDA-CA-AKI) trial with the use of short-term, high-dose atorvastatin pretreatment.^54^ While there remains a need for further evidence in support of the prophylactic use of statins to prevent CA-AKI, the majority of the patients undergoing PCI already have an indication for a high-intensity statin. Therefore, the recommendation is to start a high-intensity statin in statin-naïve patients, and in those who are already taking a statin, continue (or switch to) high-intensity statin therapy.

Finally, any nephrotoxic medications should be discontinued at least 24 to 48 hours prior to PCI. It is acceptable to continue ACEIs in patients with mild-to-moderate CKD.^55^ There is no role for dopamine, calcium channel blockers, fenoldopam, or L-arginine in the prevention of CA-AKI, while diuretics and mannitol could further worsen renal function. When in doubt, or in patients with severe CKD (≥ Stage 4), consultation with a nephrologist is recommended to assist with optimization of renal function prior to PCI.

## Procedural Strategies to Prevent Contrast-Associated Acute Kidney Injury

Minimizing contrast volume is the single most important procedural strategy to reduce CA-AKI after PCI. This strategy starts at procedure “time-out” with the cath lab team involved in the case recognizing the patients’ baseline creatinine, eGFR, and maximum allowable contrast dose (MACD). MACD can be easily calculated using the formula 5 mL x body weight (kg)/serum creatinine (mg/dL), with a maximum dose of 300 mL. Incremental use of contrast beyond the MACD is associated with a significantly increased risk (average of 45% higher risk, OR 1.45; 95% CI, 1.29–1.62) of CA-AKI.^56^

Increasing complexity of PCI has translated to higher contrast volumes used per procedure. At the same time, it is often not feasible to stop in the middle of a procedure because the MACD has been exceeded. Fortunately, more advanced techniques and technologies are available that can be leveraged to help offset this. See Table 2 for a list of contrast-sparing strategies that should be adopted for all PCIs where possible but especially when CKD is present.

**Table 2:** Contrast-sparing strategies recommended for all percutaneous coronary interventions when possible but especially in the presence of chronic kidney disease.


Angiography	Retrieve previous (and recent) diagnostic coronary angiograms to avoid repeat acquisition.

	Use high frame rate (eg, 30 frames/s) acquisitions to improve image quality (at the cost of higher radiation dose).

	Consider biplane angiography.

Guiding catheters	Avoid side-holes in guide catheters where possible.

	Avoid test injections with contrast to determine guide catheter engagement. Instead, use a coronary wire or inject normal saline (EKG repolarization changes confirm guide engagement).

Contrast media and volume	Use iso-osmolal or low-osmolal contrast media (In practice, high-osmolal agents are rarely if ever used for coronary angiography).

	Limit or eliminate the volume of contrast per injection (2 mL/injection).

	Use automated contrast injectors.

	Use diluted (with 50% normal saline) contrast media.

	Eliminate contrast in the guide catheter by back bleeding prior to administration of medications or advancing equipment.

	In high-risk patients, consider the use of newer devices that minimize contrast injection volumes or divert contrast from the kidney (see details below).

Vessel wiring, lesion assessment, stent deployment and optimization	Use a previously performed coronary computed tomography angiogram to create a live road map of the coronary tree for guidewire navigation (Syngo Fusion, Seimens Healthcare; SmartCT Roadmap, Phillips).

	Wire side branches (metallic roadmap) to aid optimal stent positioning.

	Use instant wave-free ratio to evaluate the hemodynamic significant of the lesion(s) (ie, is intervention truly needed?).

	Use intravascular ultrasound or dextran-based optical coherence tomography (experimental) to locate and assess lesions, identify proximal and distal stent landing zones, and confirm adequate stent expansion and apposition.

	Use stent enhancement technologies to position balloons and confirm adequate expansion (ClearStent, Siemens Healthcare; StentBoost, Philips; Intrasight Device Detection, Philips).


For patients with advanced CKD who require revascularization, Ali et al. have taken a further step to demonstrate the feasibility and safety of “zero contrast” PCI. This is achieved using a combination of the strategies above but with an extra emphasis on intravascular ultrasound (IVUS) guidance and pre- and post-PCI measurements of fractional flow reserve or iFR to confirm physiological improvement and success of the procedure.^57^ “Zero contrast” PCI was performed 1 week after diagnostic angiography, and this approach resulted in successful PCI with no MACE and preservation of renal function within a median follow-up time of 79 days. The average creatinine was 4.2 g/dL with an eGFR of 16 ± 8 mL/min/1.73 m^2^. Since then, larger studies have been conducted by Rahim et al. and Shibata et al., which both consistently showed that “zero contrast” PCI may be safely performed even in complex lesions with high procedural success and minimal complications, with satisfactory acute and long-term renal and cardiovascular outcomes.^58,59^

With regard to contrast media type, high-osmolar contrast media should always be avoided. However, at present, there is no evidence to recommend iso-osmolar over low-osmolar contrast media for the prevention of AKI. A recent meta-analysis by Pandya et al. from 10 randomized trials that included patients with CKD stage 3 undergoing coronary angiography demonstrated no significant difference in the incidence of CA-AKI between the use of iso-osmolar or low-osmolar contrast media.^60^ Similar lack of benefit was seen in a large observational study by Azzalini et al.^61^

Dedicated devices to reduce contrast volume administration are slowly becoming standard of care. The most prevalent are automated contrast injectors (ACIs), which can deliver less contrast volume while still maintaining optimal image quality. A recent meta-analysis by Minisinger et al. that included 79,694 patients from 10 studies showed that, on average, ACIs significantly reduced the volume of contrast delivered by up to 45 mL/case (95% CI, -54 to -35, *P* < .001) compared with manual injection while also significantly reducing the incidence of CA-AKI by up to 15% (OR 0.85; 95% CI, 0.78- 0.93, *P* < .001).^62^

The DyeVert PLUS system (Osprey Medical) also is able to reduce the volume of contrast delivered by up to 15.5% (85.6 ± 50.5 mL vs 101.3 ± 71.1 mL; *P* = .02) by diverting excess contrast in the aortic root that would not contribute to coronary opacification. However, the trial was unable to show a meaningful difference in the incidence of CA-AKI.^63^ More research is needed to determine if a newer generation of this device would translate into a clinically meaningful benefit.

Intravascular imaging is the stalwart of contrast-sparing PCI. This goal is best achieved with IVUS, which can help localize coronary lesions, identify stent landing zones, and confirm adequate stent expansion and apposition with little to no contrast at all. In the Minimizing Contrast Utilization with IVUS Guidance in Coronary Angioplasty (MOZART) trial, Mariani et al. demonstrated that IVUS-guided PCI is safe and markedly reduced contrast utilization (20 mL [12.5-30 mL] in the IVUS group versus 64.5 mL [42.8-97 mL] in the angiography group; *P* < .001) compared with angiography guidance alone.^64^ While there was no appreciable difference in the incidence of CA-AKI between both groups, this was a pilot trial with a small sample size. The ongoing MOZART II trial (NCT02743156) has been designed to study this further and will likely cement the use of IVUS during PCI to further reduce contrast volume in patients at high risk for CA-AKI.

The majority of available evidence supports the use of radial access over femoral access in patients undergoing PCI to reduce the risk of CA-AKI. It is postulated that the lower major bleeding rates seen with radial access reduce hemodynamic disturbances that would further compromise renal perfusion and lead to subsequent kidney injury. The radial approach also may reduce atheromatous embolization to the renal arteries by completely avoiding manipulation of the abdominal aorta when advancing catheters. The benefit of radial access in decreasing the risk of CA-AKI has been demonstrated in a meta-analysis of six observational studies (OR 0.51; 95% CI, 0.39-0.67, *P* < .001). Similar findings also have been demonstrated in the MATRIX-Access trial, which randomized 8,210 patients with ACS undergoing PCI to radial versus femoral access. Radial access was superior to femoral access in decreasing the risk of CA-AKI (15.4% vs 17.4%; OR 0.87; 95% CI, 0.77-0.98; *P* = .02).

Hemodynamic support with an Impella, especially in patients with hemodynamic instability, cardiogenic shock, acute heart failure, and/or CKD, is a promising strategy to reduce CA-AKI in patients undergoing high-risk PCI. The Impella can reduce the episodes of transient hypotension associated with prolonged balloon inflation times (eg, with intravascular lithotripsy or kissing balloon inflations) or no reflow following atherectomy that decrease renal perfusion and cause repetitive insults to the kidneys, which can trigger AKI. In a retrospective study, Flaherty et al. demonstrated that Impella support is protective against AKI during high-risk PCI. The incidence of AKI following high-risk PCI in patients with an ejection fraction < 35% regardless of baseline kidney function is lower with an Impella device than without (5.2% versus 27.8%; *P* < .001), and this is especially true in those patients who had existing CKD.^65,66^ Additionally, in Impella-supported patients, there was no association between the severity of CKD and the incidence of CA-AKI. By contrast, the incidence of CA-AKI was greater in unsupported patients with CKD, which was also correlated with the severity of CKD. While further exploration of these findings is warranted to validate this renal protection strategy, these data support a new renal protective strategy to reduce CA-AKI during high-risk PCI.

## Conclusion

CA-AKI remains a major complication following PCI, especially in patients with CKD. Prevention is the best and only option to avoid the significant morbidity and mortality that is associated with CA-AKI. This starts with risk assessment to identify patients at high risk of CA-AKI and is followed by implementation of pre-procedural strategies (namely hydration) and procedural strategies (namely contrast-sparing strategies) to minimize the risk of CA-AKI in all patients undergoing PCI. There is evidence to promote the use of automated contrast injectors, intravascular imaging, radial access, and—in the highest-risk patients—hemodynamic support (with an Impella). While future ongoing studies will validate these strategies, we should continue to implement best practices with what is known today, including the implementation of systemwide evidence-based case bundles to reduce CA-AKI and improve outcomes in this vulnerable patient population.

## Key Points

- Contrast-associated kidney injury is associated with a high rate of death.
- Scoring systems exist to predict an individual’s risk of developing contrast-associated kidney injury.
- Published protocols guide hydration to prevent kidney injury in patients at risk for contrast-associated kidney injury.
- The use of automated injection devices should be considered to limit the amount of contrast used on coronary angiography.

## CME Credit Opportunity

Houston Methodist is accredited by the Accreditation Council for Continuing Medical Education (ACCME) to provide continuing medical education for physicians.

Houston Methodist designates this Journal-based CME activity for a maximum of *1 AMA PRA Category 1 Credit*™. Physicians should claim only the credit commensurate with the extent of their participation in the activity.

Click to earn CME credit: learn.houstonmethodist.org/MDCVJ-18.4.
